# Proteomic alterations associated with residual disease in neoadjuvant chemotherapy treated ovarian cancer tissues

**DOI:** 10.1186/s12014-022-09372-y

**Published:** 2022-10-04

**Authors:** Emily R. Penick, Nicholas W. Bateman, Christine Rojas, Cuauhtemoc Magana, Kelly Conrads, Ming Zhou, Brian L. Hood, Guisong Wang, Niyati Parikh, Ying Huang, Kathleen M. Darcy, Yovanni Casablanca, Paulette Mhawech-Fauceglia, Thomas P. Conrads, G. Larry Maxwell

**Affiliations:** 1 Women’s Health Integrated Research Center, Gynecologic Cancer Center of Excellence, Department of Gynecologic Surgery and Obstetrics, Uniformed Services University of the Health Sciences, Walter Reed National Military Medical Center, 8901 Wisconsin Avenue, Bethesda, MD 20889 USA; 2 Murtha Cancer Center Research Program, Department of Surgery, Uniformed Services University of the Health Sciences, Walter Reed National Military Medical Center, 8901 Wisconsin Avenue, Bethesda, MD 20889 USA; 3 Henry M. Jackson Foundation for the Advancement of Military Medicine, Inc., 6720A Rockledge Dr., Suite 100, Bethesda, MD 20817 USA; 4 Department of Anatomic Pathology, Division of Gynecologic Pathology, University of Southern California, Los Angeles, CA 9007 USA; 5 Women’s Health Integrated Research Center, Women’s Service Line, Inova Health System, 3289 Woodburn Rd, Falls Church, VA 22003 USA

**Keywords:** Ovarian cancer, Neoadjuvant chemotherapy, Residual disease, Proteomics, Personalized medicine, Biomarkers

## Abstract

**Background:**

Optimal cytoreduction to no residual disease (R0) correlates with improved disease outcome for high-grade serous ovarian cancer (HGSOC) patients. Treatment of HGSOC patients with neoadjuvant chemotherapy, however, may select for tumor cells harboring alterations in hallmark cancer pathways including metastatic potential. This study assessed this hypothesis by performing proteomic analysis of matched, chemotherapy naïve and neoadjuvant chemotherapy (NACT)-treated HGSOC tumors obtained from patients who had suboptimal (R1, n = 6) versus optimal (R0, n = 14) debulking at interval debulking surgery (IDS).

**Methods:**

Tumor epithelium was harvested by laser microdissection from formalin-fixed, paraffin-embedded tissues from matched, pre- and post-NACT treated tumors for twenty HGSOC patients and analyzed by quantitative mass spectrometry-based proteomics.

**Results:**

Differential analysis of patient matched pre- and post-NACT treated tumors revealed proteins associated with cell survival and metabolic signaling to be significantly altered in post-NACT treated tumor cells. Comparison of pre-NACT treated tumors from suboptimal (R1) versus optimally (R0) debulked patients identified proteins associated with tumor cell viability and invasion signaling enriched in R1 patients. We identified five proteins altered between R1 and R0 patients in pre- NACT treated tumors that significantly correlated with PFS in an independent cohort of HGSOC patients, including Fermitin family homolog 2 (FERMT2), a protein elevated in R1 that correlated with disease progression in HGSOC patients (multivariate Cox HR = 1.65, Wald p = 0.022) and increased metastatic potential in solid-tumor malignancies.

**Conclusions:**

This study identified distinct proteome profiles in patient matched pre- and post-NACT HGSOC tumors that correlate with NACT resistance and that may predict residual disease status at IDS that collectively warrant further pre-clinical investigation.

**Supplementary Information:**

The online version contains supplementary material available at 10.1186/s12014-022-09372-y.

## Background

Ovarian cancer is the fifth most common cause of death among women in the United States, where the high-grade serous ovarian cancer (HGSOC) histologic subtype comprises approximately 95% of ovarian cancers diagnosed [1]. Standard clinical management of HGSOC has typically adhered to a paradigm characterized by primary debulking surgery (PDS) followed by platinum and taxane therapy [2]. Optimal cytoreduction consists of reducing tumor burden to no residual disease (R0) and achieving this status has been shown to significantly improve adjuvant chemotherapy response [3, 4]. Approximately 70% of stage III and IV ovarian cancer patients have wide spread disease at time of PDS, which precludes cytoreduction to R0 in greater than 80% of cases and often prompts consideration of neoadjuvant chemotherapy (NACT) [5]. Residual disease after PDS has a substantial negative impact on clinical outcomes, such as response to adjuvant chemotherapy, progression-free survival, and overall survival [6].

Neoadjuvant chemotherapy in newly diagnosed HGSOC patients followed by interval debulking surgery (IDS) offers an alternate approach to management of advanced epithelial ovarian cancer to achieve complete resection. Two prospective randomized controlled trials reported a higher R0 rate among patients who underwent NACT: 35–51% for the NACT group compared to 15–19% for the PDS group [2, 7]. Based on recent evidence, a distinction between R0 and any residual disease can guide management options [8]. Fagotti et al. proposed a laparoscopic model as an opportunity for quality improvement to predict optimal cytoreduction in advanced ovarian cancer; at a predictive index score ≥ 8 during triage laparoscopy, the probability of optimally resecting disease at primary debulking surgery is equal to 0 and the patient should receive NACT [9]. The use of NACT for patients with advanced stage epithelial ovarian cancer has steadily increased over the past decade, particularly for those patients with widespread disease.

The expression of tissue biomarkers in post-NACT ovarian samples has been evaluated and expression patterns correlated with effects on survival [10]. Additional research has identified significant cytologic as well as histologic changes that occur as a result of treatment with NACT, with cell line work in platinum sensitive and resistant ovarian cancer cells demonstrating elevations in leukocyte cell adhesion molecule (ALCAM) in resistant lines [11–13]. The proteomic profiles of paired chemotherapy naïve (pre-NACT) and chemotherapy exposed (post-NACT) ovarian tissues from patients with variations in residual disease status at IDS have not been evaluated. Therefore, the objectives of this study were to (1) determine the proteomic impact of chemotherapy in HGSOC patient-matched tissue specimens collected prior to and after neoadjuvant chemotherapy and to (2) further evaluate differences in the proteomic profiles based on optimal (R0) and suboptimal (R1) surgical debulking in pre-NACT tumors that may predict the ability to achieve optimal debulking status.

## Methods

### High grade serous ovarian cancer patient cohort and tissue specimens

High grade serous ovarian cancer patients were treated at the Keck Hospital of University of Southern California; all study protocols were approved for use under a Western IRB-approved protocol “An Integrated Molecular Analysis of Endometrial and Ovarian Cancer to Identify and Validate Clinically Informative Biomarkers” deemed exempt under US Federal regulation 45 CFR 46.102(f). All experimental protocols involving human data in this study were in accordance with the Declaration of Helsinki. Chemo-naïve tumor tissues were obtained from diagnostic biopsy whereas post-neoadjuvant treated tumor tissues were collected during IDS (Additional file 1: Fig S1). Disease assessments were evaluated and categorized based on disease distribution, residual disease status, and progression-free survival following central review of redacted medical records. Residual disease status was evaluated at the end of the IDS and was classified following review of the operative report as R0 defined as < 0.1 cm and R1 defined as ≥ 0.1–1.0 cm residual disease.

### Quantitative proteomic methods

Tumor cells were harvested from patient matched chemotherapy naïve and NACT- treated FFPE tissues by laser microdissection. Collections were processed by adding 80 µl of a buffered acetonitrile solution (100 mM ammonium bicarbonate, pH 7.63 in 20% acetonitrile) and incubated in a thermocycler at 99 °C for 1 h followed by 65 °C for 2 h. Proteins were digested by adding 1 µg of trypsin (SMART Digest Trypsin, ThermoFisher Scientific) and incubating at 50 °C overnight. Each protein digest was transferred to 0.5 mL microcentrifuge tubes, vacuum dried, and resuspended in 45 μL of LC-MS grade water. Final digest concentrations were determined by colorimetric assay (Pierce BCA Protein Assay Kit, ThermoFisher Scientific).

Samples were labeled with tandem-mass tag (TMT) isobaric labeling reagents (TMT10plex^™^ Isobaric Label Reagent Set, Thermo Fisher Scientific) according to the following procedure. First, 2.4 µg of peptide from each patient sample was pooled to create a reference standard. Next, 10 µg of peptide from each sample was aliquoted in a final volume of 100 µL of 100 µM triethyl ammonium bicarbonate. TMT labeling was then performed according to the manufacturer’s protocol with each 10-plex containing a reference standard channel (TMT 126 m/z isobaric tag) and the remaining nine TMT channels corresponding to nine individual patient samples. TMT reagents (0.8 mg) were brought to ambient temperature, dissolved in 41 μL of anhydrous acetonitrile and labeling reactions were incubated at ambient temperature for 1 h with occasional shaking. The reactions were quenched by adding 8 μL of 5% hydroxylamine followed by a 30 min incubation at ambient temperature. TMT-labeled samples were combined and vacuum dried to 82 μL. TMT-labeled peptide digests were fractionated offline (1260 Infinity II offline liquid chromatography system, Agilent) into twelve concatenated fractions. Each TMT 10 fraction was resuspended in 25 μL 100 mM NH_4_HCO_3_ and analyzed in duplicate by LC-MS/MS employing a nanoflow LC system (EASY-nLC 1200, ThermoFisher Scientific) coupled online with an Orbitrap Fusion Lumos Tribrid MS (Thermo Fisher Scientific).

Peptide identifications and protein level abundance data was assembled as recently described [14]. Differential analysis was performed using LIMMA package (version 3.8) in R (version 3.5.2) and significantly altered proteins exhibited a LIMMA p < 0.01 or a LIMMA p < 0.05 and at least a ± 1.5-fold change for a given comparison. Protein alterations were visualized by volcano plots or heatmaps and principle component analysis, with the latter methods using default settings in the ClustVis web tool [15]. Pathway analyses of altered proteins of interest was performed utilizing Ingenuity Pathway Analysis (Qiagen).

### Outcomes analysis

Protein alterations in R1 versus R0 patients were correlated with PFS in a validation cohort of 154 patients through the Clinical Proteomic Tumor Analysis Consortium (CPTAC) with a disease-free survival status of “DiseaseFree” or “Recurred/Progressed” [16]. PFS was evaluated with time from date of diagnosis to date of recurrence or progression for events and date of last contact for censored cases. Univariate and multivariate Cox regression analyses for PFS were performed for individual proteins evaluated as both categorized and continuous variables as previously described [17]. Survival distributions for PFS were generated as months from diagnosis using Kaplan–Meier methods and were compared using log-rank test. Proteins were categorized at the median with levels ≤ median expressed as low and those > median indicative of high expression. Adjusted analyses were performed controlling for age at diagnosis, residual disease, and stage. Comparison with residual disease was also performed for a subset of 138 of the 154 patients in the CPTAC cohort for which data defined under “tumor_residual” was available.

## Results

Twenty patients with pre-(chemotherapy naïve) and post-NACT (chemotherapy exposed) HGSOC tissues were included in the analysis (Additional file 1: Fig S1 and Table 1). Most patients were 51–59 years old, Hispanic, and received four cycles of platinum-based chemotherapy prior to IDS. Sixty percent of the patients had high disease distribution and 70% achieved optimal cytoreduction (R0) at IDS. Patients received standard-of-care carboplatin and paclitaxel chemotherapy regimens. Approximately 80% of patients progressed.

**Fig. 1:**
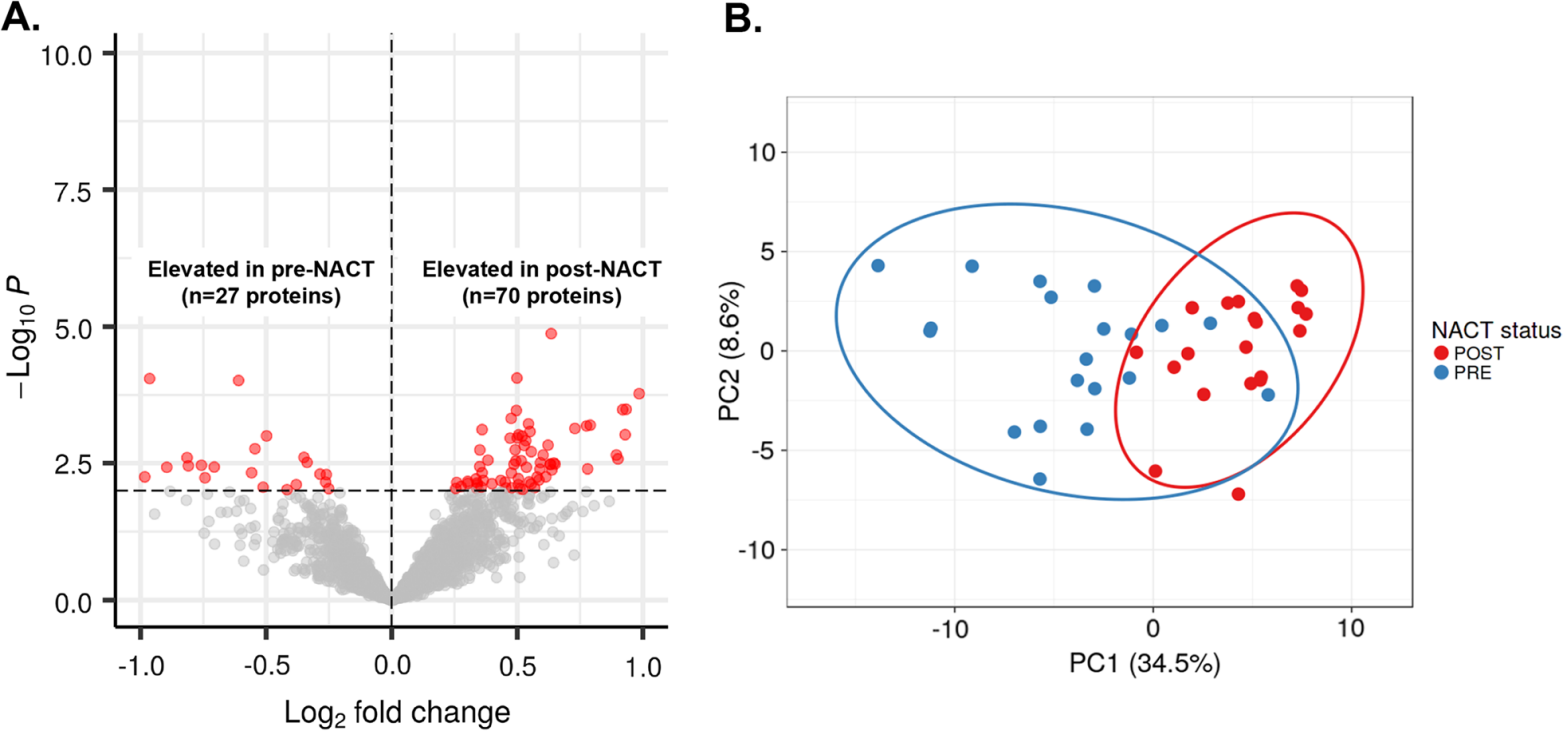
Differential analysis of quantitative proteomic data generated from matched FFPE tissues collected from HGSOC patients (n = 20), post versus pre-neoadjuvant chemotherapy (NACT) treatment. **A** Volcano plot showing 97 proteins significantly altered between post and pre-NACT treated tissues (LIMMA p < 0.01). **B** Principal component (PC) analysis of the 97 altered proteins serves explain 34.5% and 8.6% of the variance between these post and pre-NACT treated tumors

Quantitative proteomic analysis of the HGSOC patient tissue specimens identified 4336 proteins, of which 3043 were quantified across all patient samples. Differential analyses of pre- and post- NACT treated tissues revealed 97 proteins as significantly altered between post and pre-NACT groups (Fig. 1A, LIMMA, p-value ≤ 0.01, Additional file 2: Table S1) that, based on principle component analysis (PCA), explained 34.5% and 8.6% of the variance between these groups (Fig. 1B). Pathway analysis of these proteins revealed activation of cell survival and fatty acid metabolism pathways and inhibition of inflammation, cellular necrosis as well as DNA damage signaling in post versus pre-NACT treated tumor cells (Table 2). We further investigated these altered proteins relative to recently published efforts describing transcripts altered in post- versus pre-NACT treated HGSOC tumors [18]. We identified three altered proteins also identified as significantly altered at the transcript level in post versus pre-NACT treated tumors that exhibited the same abundance trends, including sorbin and SH3 domain containing 2 (SORBS2, post versus pre-NACT LogFC =  + 0.92), DNA methyltransferase 1 (DNMT1, post versus pre-NACT LogFC -0.498) and solute carrier family 2 member 1 (SLC2A1, post versus pre-NACT LogFC = − 0.964) [18].

**Table 1:** Clinical characteristics for the high grade serous ovarian cancer patient cohort (n = 20) with paired pre- and post-neoadjuvant chemotherapy (NACT) treated tumor samples analyzed by proteomics

Clinical characteristic	Case (%)
Age at diagnosis	
< 50 years old	4 (0.20)
50–59 years old	9 (0.45)
60–69 years old	4 (0.20)
70–79 years old	3 (0.15)
Race and ethnicity	
White	3 (0.15)
Black	4 (0.20)
Hispanic	13 (0.65)
Stage	
III NOS	3 (0.15)
IIIB	1 (0.05)
IIIC	5 (0.25)
IV NOS	3 (0.15)
IVA	3 (0.15)
IVB	5 (0.25)
Residual disease	
R0	14 (0.70)
R1	6 (0.30)
Disease distribution^a^	
Moderate	8 (0.40)
High	12 (0.60)
Cycles of NACT paclitaxel and carboplatin	
3	6 (0.30)
4	13 (0.65)
6	1 (0.05)
Recurrence^b^	
Yes	16 (0.80)
No	4 (0.20)

^a^Disease distribution was classified as low when disease was limited to the pelvic cavity and retroperitoneal lymph nodes; moderate for cases with pelvic, retroperitoneal and abdominal spread sparing the upper abdomen; high when disease spread to the upper abdomen including the diaphragm, liver, spleen, or pancreas

^b^Median follow-up was 1.9 years with a range of 0.53 to 9.09 years.

**Table 2:** Top pathways activated or inhibited in post versus pre-NACT treated tumors

Diseases or functions annotation	Post versus Pre-NACT^a^ (Activation z-score)
Synthesis of lipid	2.586
Cell survival	2.327
Cell viability	1.926
Phagocytosis of cells	1.671
Synthesis of fatty acid	1.534
Quantity of hydrogen peroxide	− 1.982
Inflammation of absolute anatomical region	− 2.023
Extracranial solid tumor	− 2.09
Inflammation of organ	− 2.118
Inflammation of body cavity	− 2.701

^a^Positive values represent activation in post-NACT ovarian cancer tissue specimens and negative values represent activation in pre-NACT ovarian cancer tissue specimens

**Fig. 2:**
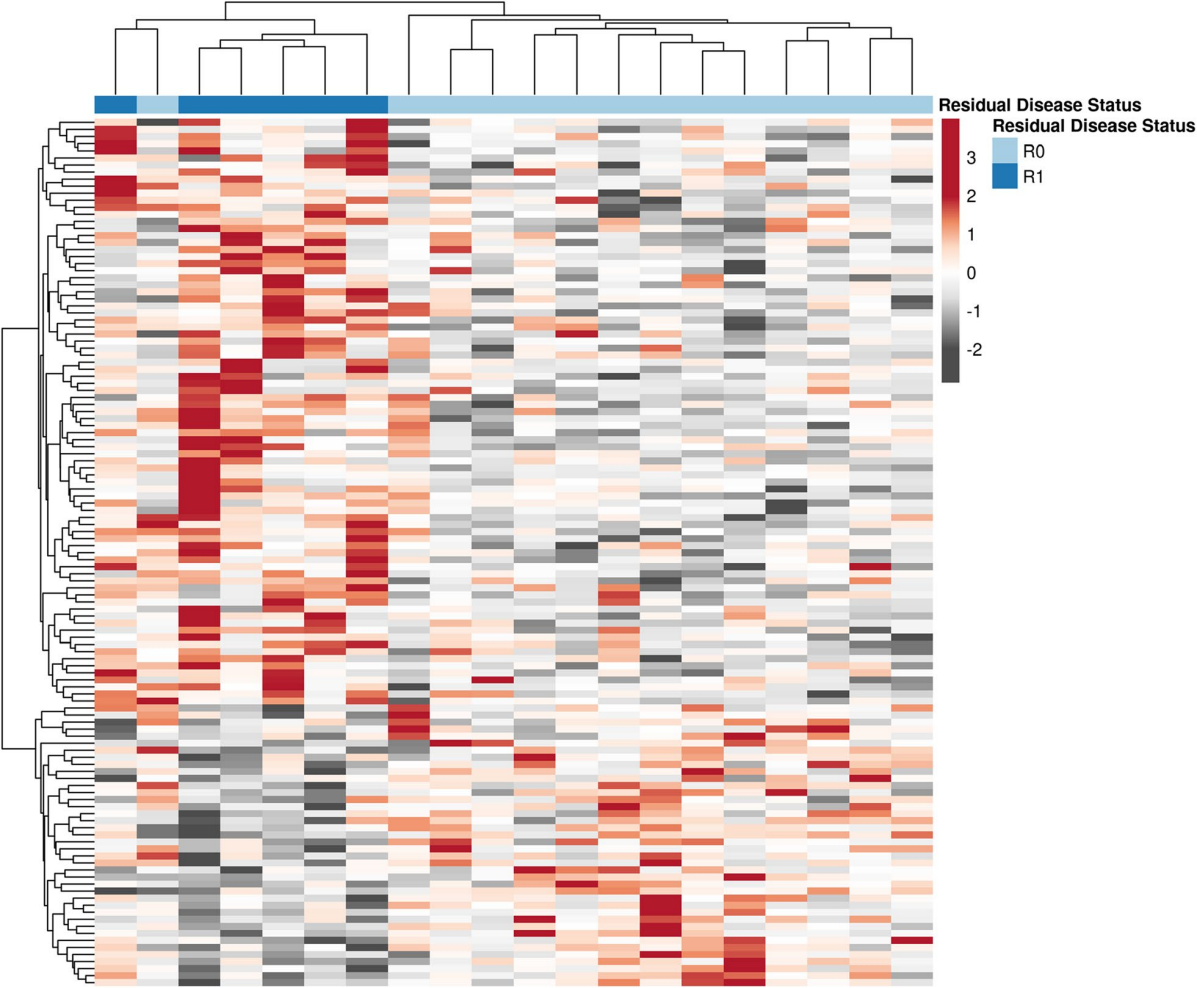
**A** Differential analysis of quantitative proteomic data generated from FFPE tissues collected pre-neoadjuvant chemotherapy (NACT) treatment from HGSOC patients with residual (R1, n = 6) or no/microscopic residual (R0, n = 14) disease at interval debulking surgery. Differential analyses of pre-NACT treated tissue revealed 140 proteins as significantly altered between these patient populations (LIMMA p-value ≤ 0.05). “R0” = none or microscopic, “R1” = macroscopic, i.e. < 1 cm disease

A subset of patients in this study had some level of residual disease following IDS (≥ 0.1–1.0 cm residual disease, R1, n = 6) versus the majority that did not (no/microscopic, < 0.1 cm residual disease, R0, n = 14), and we compared differential residual disease status in pre-NACT (chemo-naïve) tumors from these patients. Differential analyses of pre-NACT tumors in R1 versus R0 patients identified 140 significantly altered proteins (Fig. 2, LIMMA p ≤ 0.05, Additional file 2: Table S2); 50 of which were substantially altered (± 1.5-fold change). We investigated these altered proteins in a public, global proteomic dataset of 154 HGSOC patients [16] comparing tumors from chemo-naïve HGSOC patients with high (> 1.0 cm, i.e. > R1, n = 50) or low (> 0.1–1.0 cm, n = 64) residual disease or with no residual disease (R0, n = 24) following debulking surgery. We identified 16 proteins that were significantly (LIMMA p < 0.05) co-altered between pre-NACT tumors from patients with any level of residual disease vs R0 patients from this independent cohort that exhibit highly correlated abundance trends with the present cohort (Spearman R = 0.645, P = 0.0069), and 6 of these proteins were substantially altered (± 1.5-fold) in patients with any level of residual disease vs R0 patient tumors (Additional file 2: Table S3). Pathway analyses of these proteins revealed activation of signaling regulating tumor cell viability, as well as cell migration and invasion and inhibition of pathways regulating generation of reactive oxygen species and bleeding in pre-NACT tumors from R1 versus R0 patients (Additional file 2: Table S4).

**Fig. 3:**
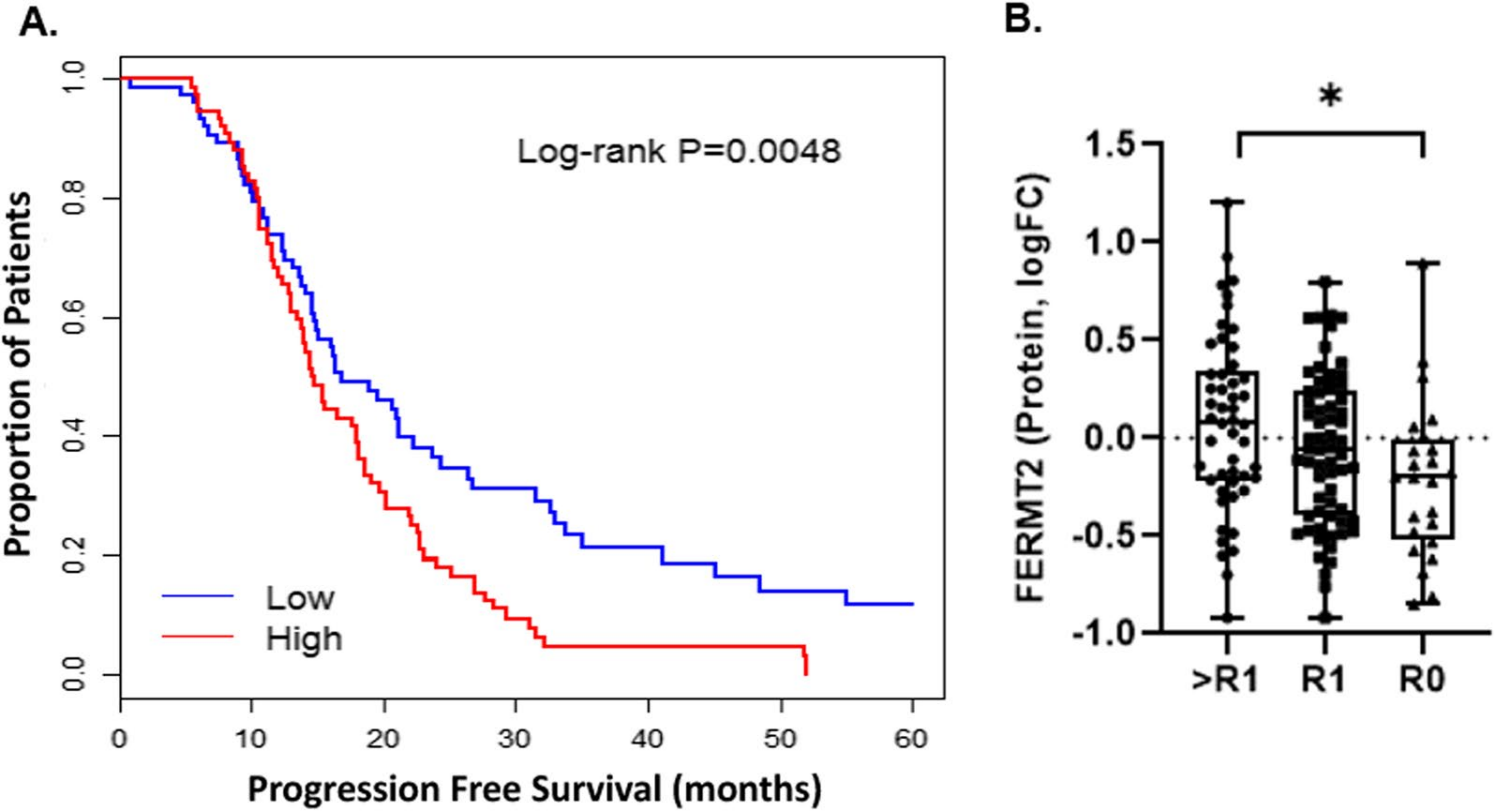
Fermitin Family Member 2 (FERMT2) correlates with an increased risk of progression and is significantly elevated in HGSOC patients with high (> 1.1 cm) residual disease burden. **A** Kaplan–Meier curve illustrating the relationship between FERMT2 protein abundance and progression free survival (PFS) identified from a publicly available global proteomics data set of 154 HGSOC patient tissues (CPTAC Ovarian, 2016); high and low reflects median cut-point of FERMT2 protein abundance and Log-rank P reflects categorized, univariate log-rank testing. **B** FERMT2 protein abundance for patients exhibiting > 1.0 cm (> R1, n = 50), 0.1–1.0 cm (R1, n = 64) or no residual disease (R0, n = 24); * reflects Mann Whitney U P-value = 0.02

To investigate if proteins altered in patients with residual disease further correlated with disease prognosis, we correlated proteins altered between R1 versus R0 patients with proteins significantly associated with progression free survival (PFS) in a public dataset of 154 HGSOC patients (univariate, Log-Rank and Wald p < 0.05) [16]. We also assessed if proteins significantly associated with disease progression did so independently of patient age, disease stage and residual disease status to identify proteins that may have an increased likelihood of influencing tumor cell biology rather than co-trending with clinical variables known to correlate with disease outcome [19]. Of the 49 proteins significantly altered in pre-NACT tumors collected from R1 versus R0 patients, four were also correlated with differential PFS, including Glutathione Synthetase (GSS, HR = 1.45, p = 0.037), Fermitin Family Member 2 (FERMT2, HR = 1.66, p = 0.005), Perilipin 2 (PLIN2, HR = 1.58, p = 0.01) and Methylthioribose-1-phosphate isomerase (MRI1, HR = 0.7, p = 0.049) (Fig. 3A, Additional file 1: Fig. S2, and Additional file 2: Table S5). FERMT2 remains significantly associated with PFS following adjustment for patient age at diagnosis, residual disease status as well as disease stage (adjusted HR = 1.65, Wald P = 0.022). FERMT2 is also among proteins significantly elevated in patients with high residual (> R1, n = 50) versus no residual (R0 n = 24) disease in an independent HGSOC cohort and further analysis shows FERMT2 is significantly elevated in R1 versus R0 patients (Fig. 3B). All proteins were elevated in R1 versus R0 patient tumors and associated with a significantly increased risk of disease progression, except MR1I (HR = 0.7, Log-Rank p = 0.049).

## Discussion

This study identified 97 proteins significantly altered between patient matched pre-NACT (chemotherapy naïve) and post-NACT (chemotherapy treated) HGSOC tumor tissue. Pathway analysis of these alterations revealed activation of pathways associated with cell survival and metabolism in post NACT treated tumors. Aldo–keto reductase family 1 member B1 (AKR1B1) is elevated in post-NACT treated tumors and correlates with alterations in fatty acid metabolism. Notably, AKR1B1 strongly correlates with epithelial-to-mesenchymal transition (EMT) and is linked to glucose metabolism, cancer differentiation, and aggressiveness [20]. Inhibition of AKR1B1 has also been evaluated in diabetes management with inhibition of AKR1B1 being shown to prevent advanced glycation end-product accumulation and atherosclerotic lesion formation [21]. Additionally, succinate-semialdehyde dehydrogenase, mitochondrial (ALDH5A1), which is involved in glutamate metabolism, was elevated in post-NACT treated tumors and work by Tian and colleagues identified that low expression of ALDH5A1 is associated with worse overall survival in ovarian cancer [22]. In our study, elevated levels of AKR1B1 and ALDH5A1 in post-NACT treated tumors suggests alterations in proteins regulating cellular metabolism accompanies exposure of ovarian cancer cells to cytotoxic chemotherapy. In support of this finding, recent evidence has shown that HGSOC tumor cells exhibit altered cellular metabolism following multiple lines of chemotherapy treatment [23]. We further observe sorbin and SH3 domain containing 2 (SORBS2) as significantly elevated in post-NACT tumors, which has also been described as elevated at the transcript level in post versus pre-NACT treated HGSOC tumors [18]. Interestingly, SORBS2 has been shown to suppress metastatic spread and to promote a tumor-suppressive microenvironment in ovarian cancer models in vivo [24].

We further compared pre -NACT treated tumors from patients with residual disease following IDS (≤ 0.1–1.0 cm residual disease, R1, n = 6) versus those with no/microscopic (< 0.1 cm) residual (R0, n = 14). Notably, pathways regulating cellular invasion and migration were predicted as activated in pre-NACT treated tumors from patients with (R1) versus without residual disease (R0) following IDS. Investigation of proteins altered among invasion and migration signaling pathways revealed branched chain amino-acid transaminase 1 (BCAT1) as elevated in pre-NACT tumors from R1 versus R0 patients (+ 1.11 LogFC, P = 0.021). BCAT1 has been shown to play a key role in promoting tumor cell invasion and migration in a variety of solid tumor malignancies including hepatocellular carcinoma, non-small cell lung cancer as well as ovarian cancer [25–27]. Silencing BCAT1 in ovarian cancer cells results in decreased proliferation, invasion, and migration and correlates with prolonged survival time in xenograft models [27]. We further investigated proteins altered between R1 vs R0 patients from pre-NACT tumors in patients with (> 0.1 cm) or without residual disease in an independent cohort of HGSOC patients (n = 154) and identified 16 proteins significantly co-altered that are quantitatively correlated with the present investigation. Although seemingly low, many important factors including differences in sample type (e.g., fresh-frozen in CPTAC and FFPE in our study), sample preparation (e.g., bulk tumor from broad differences in tumor purity in CPTAC and LMD enriched tumor epithelium in our study), and patient cohort size, likely impact the overlap of significantly altered proteins observed in our study compared to previously published work.

To investigate if proteins altered in patients with residual disease also correlate with disease prognosis, we evaluated the relationship of proteins altered between R1 versus R0 patients with proteins associated with PFS in a publicly available cohort of global proteomic data from 154 HGSOC patients [16]. We further assessed if proteins significantly correlating with PFS did so independently of patient age, disease stage and residual disease status as these clinical variables are known to correlate with disease outcome [19]; residual disease is a particularly significant prognostic factor determining ovarian cancer related outcome and it also has bias associated with experience of the surgeon and complexity of the surgery tolerated by the patient. Our goal for this analysis was to prioritize proteins that may have an increased likelihood of influencing tumor cell biology resulting in altered invasive and metastatic potential that could in part contribute to higher disease burden. We identified proteins significantly altered in R1 versus R0 tissues that correlate with PFS; Glutathione Synthetase (GSS), Fermitin Family Member 2 (FERMT2), Perilipin 2 (PLIN2) and Methylthioribose-1-phosphate isomerase (MRI1) were altered in pre-NACT tumors. GSS and FERMT2 were also among proteins significantly elevated in patients with (> 0.1 cm) or without (no macroscopic) residual disease in an independent cohort of 154 tumors from chemo-naïve HGSOC patients. GSS, which we observed to be associated with an increased risk of disease progression, is an enzyme involved in the glutathione biosynthesis pathway and has been described as a potent antioxidant protecting cells from oxidative damage by free radicals and detoxification [28]. FERMT2 was upregulated in R1 versus R0 chemotherapy naïve tissues as well as in an independent cohort of HGSOC patients with (> 0.1 cm) versus without (no macroscopic) residual disease (n = 154), and that correlated with an increased risk of disease progression independent of patient age at diagnosis, residual disease status as well as disease stage. FERMT2, also known as Kindlin-2, has been associated with poor survival in lung adenocarcinoma [29] and hepatocellular carcinoma [30] and FERMT2 as well as other fermitin family members have been shown to promote EMT and metastasis through the ß catenin pathway [31–33]. In contrast, we observed increased abundance of MRI1 in pre-NACT tissue samples from R1 vs R0 patients and this protein was also correlated with a lower risk of disease progression. MRI1 plays an essential role in methionine formation, which in turn functions in protein translation, methylation, and protection against reactive oxygen species through the generation of glutathione [34, 35]. As MRI1 is related to promoting glutathione signaling, and as noted above for GSS, the specific role of MRI1 in decreasing the risk of disease progression in ovarian cancer will require further investigation to understand whether this protein promotes tumor cell survival through mitigating oxidative stress. Notably, both GSS and MRI1 did not remain significantly correlated with PFS following adjustment for common covariates of disease progression, such as patient age, disease stage and residual disease status. Perilipin 2 (PLIN2) was correlated with an increased risk of disease progression and was elevated in in patients with (R1) or without (R0) residual disease in pre-NACT tissues. The PLIN family are associated with controlling lipolysis [36]. Research by Liu et al. demonstrates that PLIN2 binds not only to lipids but other Wnt signaling components, with depletion in the presence of lipids affecting the normal association of PLIN2 [36]. The Wnt/ß-catenin pathway is one of the major signaling pathways thought to be involved in EMT, with alterations in this pathway affecting Wnt pathway proteins on the cell membrane, cytoplasm, and nucleus [37]. Additional work by Arend et al. [37] evaluated niclosamide, an FDA-approved derivative for treating tapeworm infections, as a target of the Wnt/ ß-catenin pathway in ovarian cancer cells. Use of niclosamide, along with two synthetically manufactured niclosamide analogs, significantly inhibited proliferation in two chemoresistant ovarian cancer cell lines, demonstrating the potential of drug repurposing for chemoresistant epithelial ovarian cancer [38]. These proteins may serve as biomarkers to stratify patients for whom optimal cytoreduction is not achievable, to provide prognostic information, or serve as future drug targets.

Among the strengths of this study includes the use of human ovarian tumors with chemo-naïve and NACT/chemotherapy-treated tumors collected from the same patient. This novel sample set allows for documenting in situ changes that occur with administration of NACT in a clinical setting. Our study is limited by a small sample size (n = 20), the uneven distribution of patients with residual disease (n = 14 with R0 and n = 6 with R1), and limited clinical data regarding patient outcomes. Another limitation is that many of our patients were predominantly of Hispanic race and thus is not necessarily representative of all populations.

The differences demonstrated between chemotherapy naïve (e.g. pre-NACT) and chemotherapy exposed (e.g. post-NACT) tissues represents a unique opportunity to investigate candidate molecular marker(s) to stratify patients with high disease distribution or who may not respond well to conventional chemotherapy. In addition, identification of adaptive tumor changes may also inform preclinical investigation of interventions aimed at overcoming resistance to NACT. Our identification of a protein signature associated with R0 debulking status in the pretreatment setting was another exciting observation with potential clinical utility. A test predictive of R0 at diagnostic laparoscopy could better inform patient counselling at diagnosis as well as forecast whether > 3 cycles of chemotherapy may be needed to optimize the chance of achieving R0 at IDS. Our exploratory efforts have not only identified proteins associated with aggressive tumor biology, but also set the stage for follow-up investigation of a patient cohort that has had more regimented decision making in selection of patients for primary cytoreduction versus NACT. Validation of our exploratory findings and identification of additional targets could enable development of a predictive signatures for ovarian cancer patient surgical outcomes.

## Conclusions

This study identified significant proteomic alterations in matched, chemotherapy naïve and NACT-treated patients tumors obtained from HGSOC patients with suboptimal (R1) versus optimal (R0) debulking at IDS. Distinct proteome profiles are present in pre-NACT HGSOC tumors correlating with residual disease status that may represent predictive biomarkers of residual disease at IDS, as well as proteins associated with NACT resistance warranting further pre-clinical investigation.

## Supplementary Information

**Additional file 1: Figure S1.** Clinical model to determine surgical algorithm for ovarian cancer patients. **A** diagnostic surgery is first conducted to determine feasibility of optimal cytoreduction in women found to have ovarian cancer. If patients are determined by the surgeon to have disease burden that is likely to achieve optimally cytoreduction (R0), then patients undergo Primary Debulking Surgery (PDS). If optimal debulking is determined to be unlikely (R1), patients have tissue specimens collected at the diagnostic surgery, but otherwise cytoreduction is not attempted. These patients were subsequently referred for neoadjuvant chemotherapy (NACT) postoperatively and then scheduled for Interval Debulking Surgery (IDS) after three or more cycles of chemotherapy. **Figure S2.** Comparison of proteins significantly altered between patients with residual disease (R1) versus no residual disease (R0) in pre-NACT ovarian cancer tissue.

**Additional file 2: Table S1.** Proteins significantly altered between post versus pre-NACT tissues (LIMMA p-value<0.01). **Table S2.** Proteins significantly altered between R1 versus R0 patients in pre-NACT tissues (LIMMA p-value<0.05). **Table S3.** Significantly (LIMMA p<0.05) co-altered proteins between pre-NACT tumors from patients with any level of residual disease vs R0 patients from an independent ovarian cancer cohort (CPTAC 2016) that exhibit highly correlated abundance trends with the present ovarian cancer cohort. **Table S4.** Diseases and biofunctions (Ingenuity Pathway Analysis) significantly enriched by proteins altered between R1 versus R0 patients in pre-NACT tissues (LIMMA p-value<0.05). **Table S5.** Proteins altered in R1 versus R0 patients in pre-NACT treated tumors significantly correlating with altered progression free survival in CPTAC ovarian 2016.

## Data Availability

The mass spectrometry data have been deposited to the ProteomeXchange Consortium via the PRIDE partner repository with the dataset identifier PXD031929.
