# Supplementary material for: Proteomic alterations associated with residual disease in neoadjuvant chemotherapy treated ovarian cancer tissues
Source: Clin Proteomics. 2022 Oct 4;19:35. doi: 10.1186/s12014-022-09372-y (PMC9531351; doi:10.1186/s12014-022-09372-y)
Supplement: Supplementary file 1 — Additional file 1: Figure S1. Clinical model to determine surgical algorithm for ovarian cancer patients. A diagnostic surgery is first conducted to determine feasibility of optimal cytoreduction in women found to have ovarian cancer. If patients are determined by the surgeon to have disease burden that is likely to achieve optimally cytoreduction (R0), then patients undergo Primary Debulking Surgery (PDS). If optimal debulking is determined to be unlikely (R1), patients have tissue specimens collected at the diagnostic surgery, but otherwise cytoreduction is not attempted. These patients were subsequently referred for neoadjuvant chemotherapy (NACT) postoperatively and then scheduled for Interval Debulking Surgery (IDS) after three or more cycles of chemotherapy. Figure S2. Comparison of proteins significantly altered between patients with residual disease (R1) versus no residual disease (R0) in pre-NACT ovarian cancer tissue. [file 12014_2022_9372_MOESM1_ESM.pptx]

## Slide 1
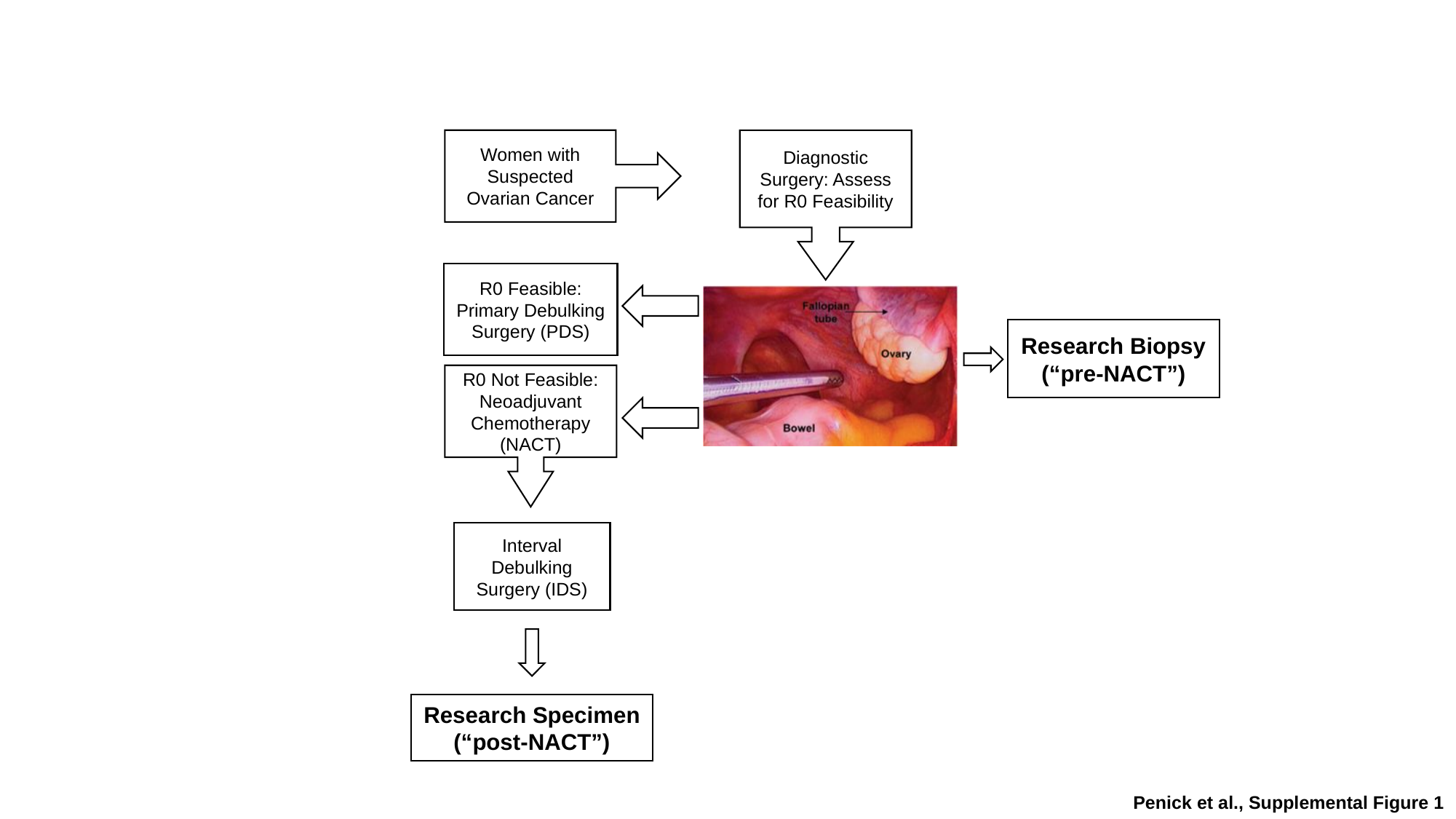

Women with Suspected Ovarian Cancer
Diagnostic Surgery: Assess for R0 Feasibility
R0 Feasible: Primary Debulking Surgery (PDS)
Research Biopsy
(“pre-NACT”)
R0 Not Feasible: Neoadjuvant Chemotherapy (NACT)
Interval Debulking Surgery (IDS)
Research Specimen
(“post-NACT”)
Penick et al., Supplemental Figure 1

## Slide 2
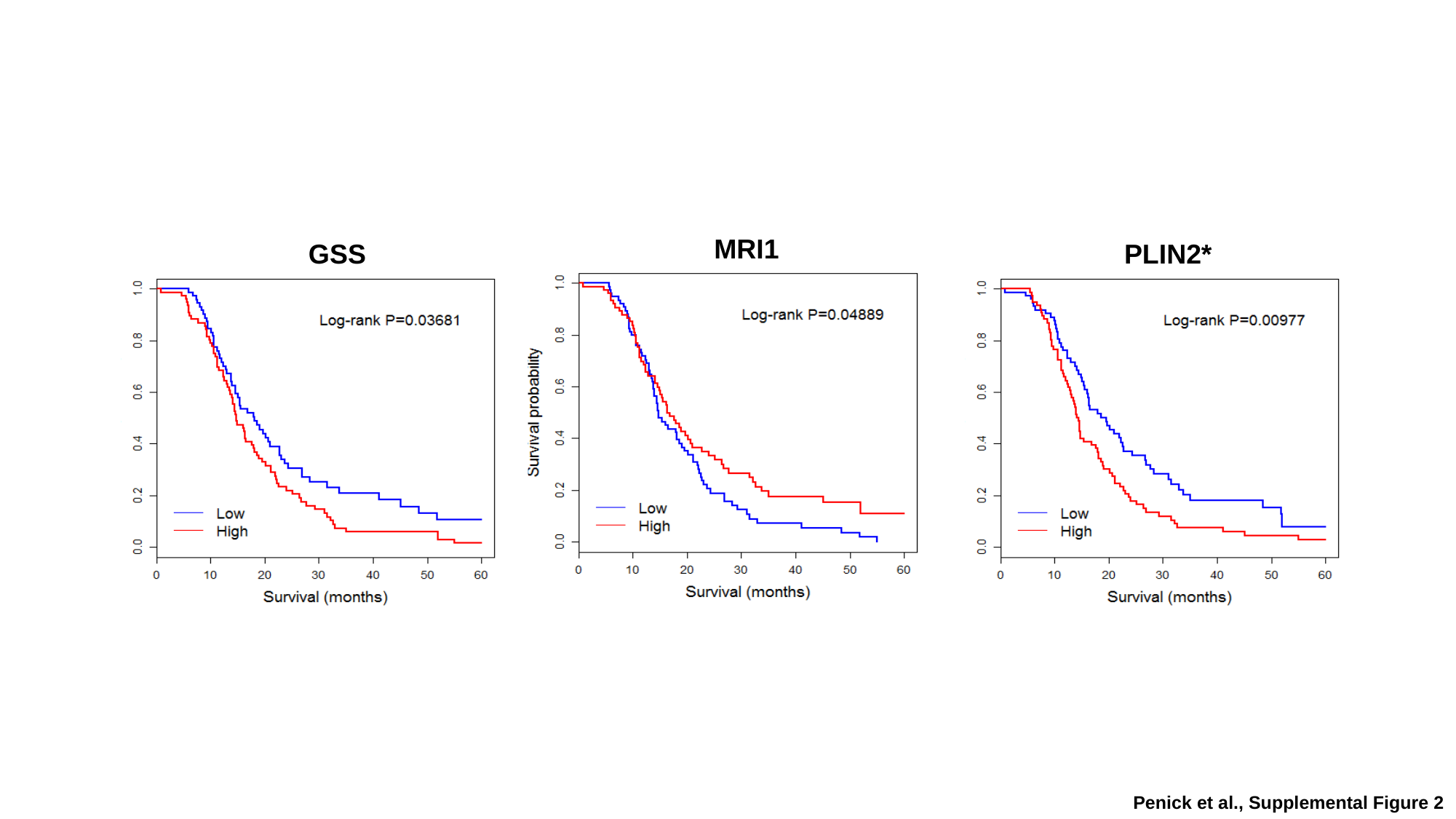

MRI1
GSS
PLIN2*
Penick et al., Supplemental Figure 2
